# Progression of mycosis fungoides in a patient receiving a Janus kinase inhibitor

**DOI:** 10.1016/j.jdcr.2025.07.019

**Published:** 2025-08-19

**Authors:** Julia M. Stankiewicz, Juliette M. Kersten, Veerle A. Merkus, Sanne de Haan, Patty M. Jansen, Maarten H. Vermeer, Rosanne Ottevanger

**Affiliations:** Department of Dermatology, Leiden University Medical Centre, Leiden, The Netherlands

**Keywords:** cutaneous T-cell lymphoma, DADA-2, JAK inhibitors, mycosis fungoides

## Introduction

Janus kinase (JAK) inhibitors are a novel type of immune-modulating medications that have recently been approved to treat various dermatological diseases, such as atopic dermatitis, alopecia areata, and vitiligo.^1^ New oral and topical JAK inhibitors are being developed and investigated in these and other cutaneous conditions. However, in 2021 the Food and Drug Administration issued a statement, warning that JAK inhibitors could increase the risk of malignancy, emphasizing the need for caution while prescribing these drugs.^2^ In addition, in the past years there have been multiple case reports and series connecting JAK inhibitors and onset or worsening of cutaneous lymphomas.^3^^,^^4^ Mycosis fungoides (MF) is the most common subtype of cutaneous T-cell lymphoma (CTCL), which follows an indolent course in most patients.^5^ In a subset of cases the disease progresses, and development of skin tumors can occur, which is linked to a poor 5-year survival. This report presents a case of progression of early-stage MF after administration of JAK inhibitors.

## Case report

**Fig 1:**
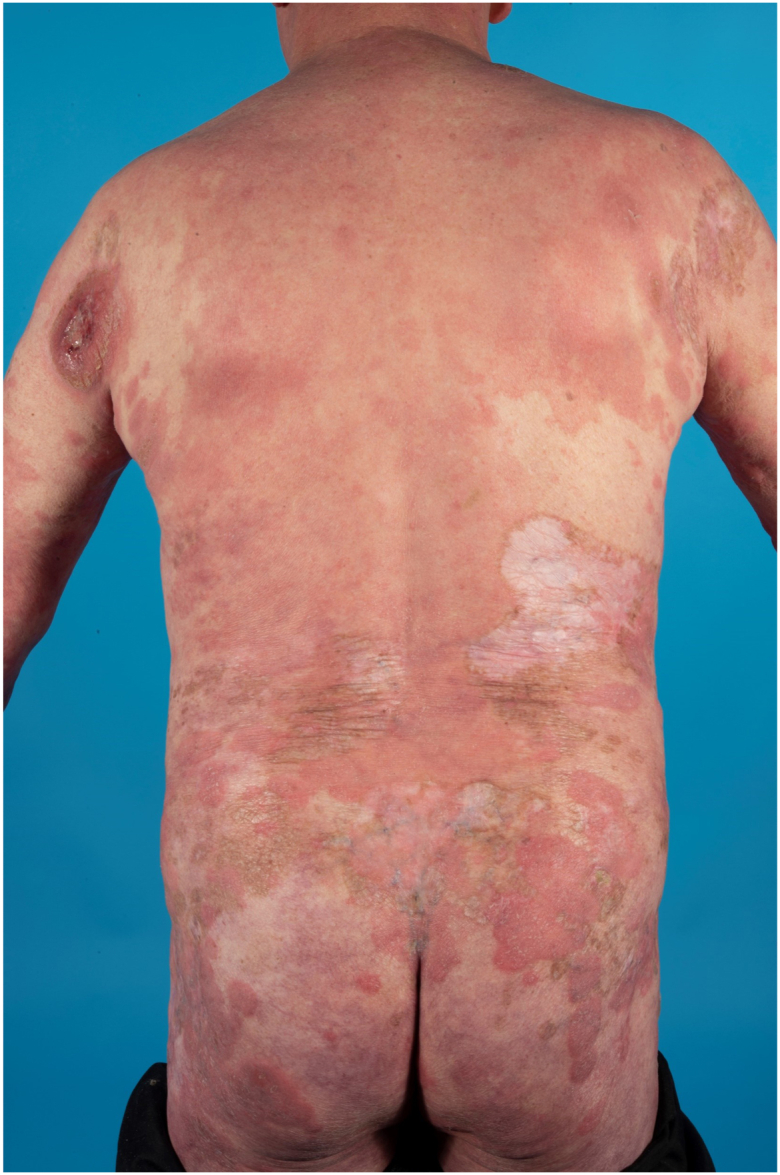
Extensive erythematosquamous plaques covering the back and buttocks. On the left shoulder a large, erosive tumor.

**Fig 2:**
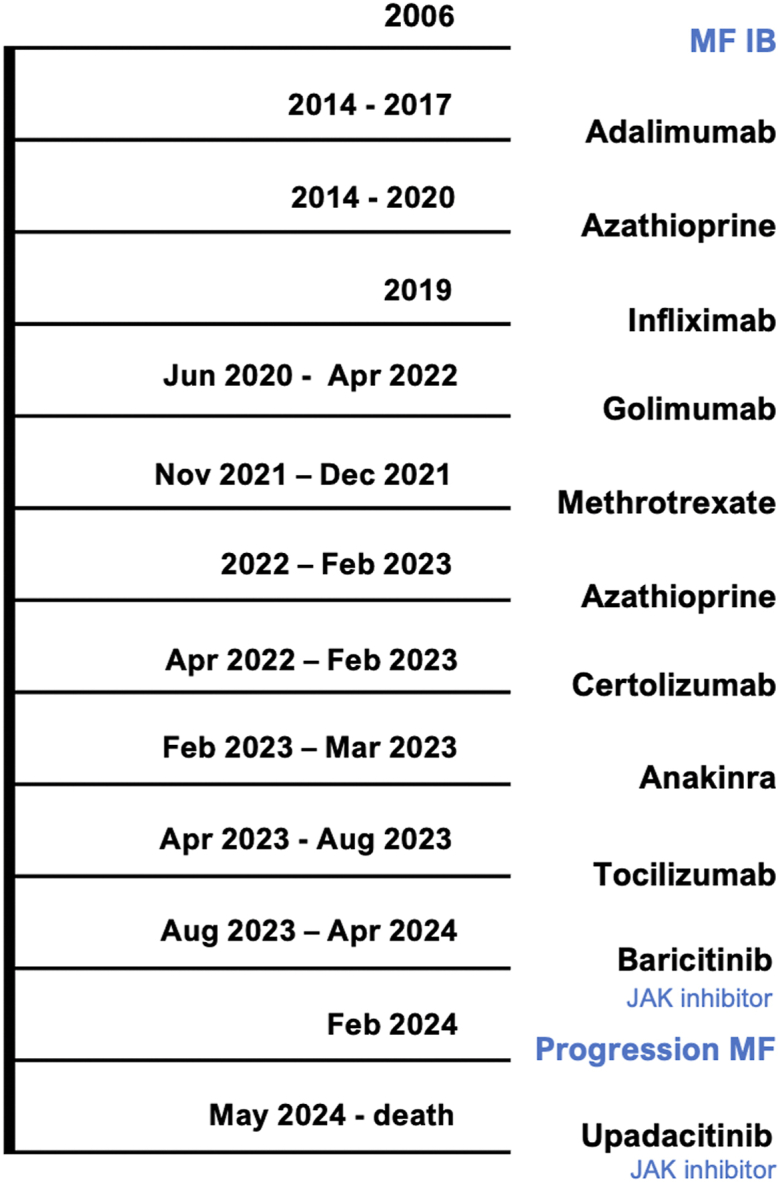
Timeline of prescribed immunomodulating therapies. *JAK inhibitor*, Janus kinase inhibitor; *MF*, mycosis fungoides.

**Fig 3:**
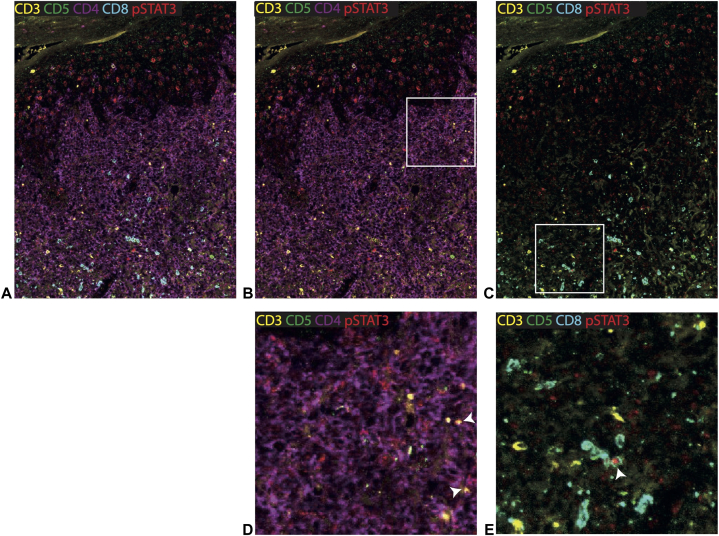
Representative multi-immunofluorescence image of a tumor biopsy highlighting phosphorylated STAT3 (pSTAT3) expression in CD8+ T cells and CD4+ tumor cells. High-resolution multispectral images were obtained on a Vectra Polaris system using a 20 × objective, yielding a spatial resolution of 0.5 μm per pixel. **(A-C)** Multiplex staining for CD3 (*yellow*), CD5 (*green*), CD4 (*purple*), CD8 (*blue*), and pSTAT3 (*red*). **A,** Full panel with all 5 markers. **B,** Selected channels highlighting CD3, CD5, CD4, and pSTAT3. The *box* indicates the region of interest (ROI) shown in panel (**D**). **C,** Selected channels highlighting CD3, CD5, CD8, and pSTAT3. The *box* indicates the ROI shown in panel (**E**). **D,** ROI from panel B. Tumor cells are defined as CD3^+^CD5^-^CD4^+^, with *arrows* indicating tumor cells expressing pSTAT3. **E,** ROI from panel C, showing CD8^+^ T cells defined as CD3^+^CD5^+^CD8^+^. *Arrow* indicates pSTAT3-expressing CD8^+^ T cell.

A 60-year-old male was referred in August 2024 to the outpatient clinic of the Leiden University Medical Center with rapid progression of infiltrated plaques and tumors on the skin (Fig 1). He had a history of MF stage IB presenting with itching patches and plaques. The past 20 years he had been treated with UVB, PUVA, and topical steroids which resulted in stable disease. Additionally, his medical history was notable for a deficiency of deaminase 2, characterized by systemic vasculitis, and immunodeficiency resulting in splenomegaly, recurrent episodes of fever, cholangitis, and recurrent cutaneous livedoid vasculitis with persistent, painful, and therapy-resistant ulcers.^6^ Since 2014 many immunomodulating therapies were prescribed to treat the vasculitis, all with minimal therapeutic effect (Fig 2). Therefore, baricitinib, a JAK inhibitor, was prescribed in August 2023. This treatment resulted in remission of the painful ulcers, vasculitis, and recurrent episodes of fever. At the start of treatment with baricitinib, his MF was stable with mostly patches covering the skin (BSA 18%, mSWAT 22). Unfortunately, in the following 7 months progression of the skin lesions was observed, with an increase in patches and plaques. An initial biopsy in May 2024 showed insufficient infiltrate to demonstrate MF involvement. However, suspected association with baricitinib resulted in its replacement by upadacitinib, also a JAK inhibitor, in May 2024. Following this change, the skin lesions progressed rapidly, eventually resulting in development of tumors and infiltrated plaques covering 80% of his body (Fig 1). Biopsies of both a tumor and a plaque, performed in August 2024, confirmed MF localization. Compared to the previous histology, an atypical lymphoid infiltrate extending into the dermis with blastoid transformation was observed. Tumor cells stained positive for CD4 and CD30, negative for CD8 and showed loss of CD5 and CD7, leading to the conclusion that a rapid progression of his MF was occurring. Positivity for phosphorylated STAT3 (pSTAT3) was detected in both CD8^+^ T cells and tumor cells (Fig 3). A positron emission tomography/computed tomography revealed not only multiple cutaneous lesions with FDG avidity, but also an enlarged lymph node (14 mm) at the level of the parotid gland, as well as several other lymph nodes showing mildly increased metabolic activity in the neck, both axillae, and both the inguinal and parailiac regions. Furthermore, 2 lesions in the lungs were observed, measuring 20 mm and 17 mm, suggestive of systemic localizations of the previously diagnosed lymphoma, resulting in stage IV MF. Lymph node biopsies were not performed as the localizations were considered not suitable for pathology sampling. Because of this aggressive course of disease, cyclophosphamide, hydroxydaunorubicin, vincristine and prednisone regimen and allogeneic stem cell transplantation were considered in multidisciplinary consultations. Unfortunately, the patient died before therapy could be administered.

## Discussion

JAK inhibitors have recently been introduced in dermatology and are positioned as a new treatment for dermatitis and alopecia areata, with ongoing research into their potential use for T-cell lymphoma.^1^^,^^7^ These agents target cells expressing JAK1, JAK2, JAK3, and TYK2, modulating the entire JAK/STAT signaling cascade, thereby preventing phosphorylation of STAT3.^8^ Consequently, cells dependent on pSTAT3 signaling are impaired in their function. In this case, treatment involved baricitinib, which targets JAK 1 and JAK 2, and later upadacitinib, which predominantly inhibits JAK 1. Both agents are known to inhibit pSTAT3 as well, with upadacitinib having a slightly stronger effect.^8^ Molecular and biomarker-driven studies demonstrate enhanced JAK/STAT signaling in CTCL tumor cells, suggesting its potential as a therapeutic target.^8^ However, multiplex immunofluorescence analysis revealed pSTAT3 expression not only in CD4+ tumor cells but also in CD8+ T-cells (Fig 3). Since these cytotoxic T-cells contribute to antitumor response, inhibiting pSTAT3 may impair their function, potentially reducing tumor immune surveillance.^9^ This mechanism may explain the rapid progression to stage IV MF observed in our patient with longstanding MF IB after administration of JAK inhibitors. Similar cases of progression of CTCL have been described, resulting in an Food and Drug Administration warning.^2^, ^3^, ^4^ Comparable effects have been described with cyclosporine, another immunomodulatory agent proposed for CTCL treatment but associated with adverse outcomes.^10^ This case and previous reports demonstrate that exploring therapeutic implications of the JAK/STAT pathway in CTCL treatment should be carefully reconsidered.^3^^,^^4^

We urge clinicians to remain cautious while prescribing JAK inhibitors, and to consider the benefits and risks for each individual patient.^2^ Particularly patients with a history of malignancies, especially in patients with (cutaneous) lymphomas, should be carefully monitored. Future research should focus on long-term adverse events and possible contraindications of JAK inhibitors, to better understand the possibilities and limitations of these novel immunomodulators.

## Conflicts of interest

None disclosed.
